# Schistosomiasis control in adults: a call for action towards the goal of universal health coverage: Sarah Gruninger

**DOI:** 10.1093/eurpub/ckac130.031

**Published:** 2022-10-25

**Authors:** S Gruninger, T Rasamoelina, R Rakotoarivelo, C Oumar Doumbia, E Lorenz, GJ van Dam, NG Schwarz, J May, R Rakotozandrindrainy, D Fusco

**Affiliations:** 1 Department of Infectious Disease Epidemiology, Bernhard Nocht Institute for Tropical Medicine, Hamburg, Germany; 2 German Center for Infection Research, Hamburg-Borstel-Lübeck-Riems, Germany; 3 Centre d’Infectiologie Charles Mérieux, University of Antananarivo, Antananarivo, Madagascar; 4 Department of Infectious Diseases, University of Fianarantsoa Andrainjato, Fianarantsoa, Madagascar; 5 University Clinical Research Center, University of Sciences Technics and Technologies of Bamako, Bamako, Mali; 6 Department of Parasitology, Leiden University Medical Center, Leiden, Netherlands; 7 Department of Microbiology and Parasitology, University of Antananarivo, Antananarivo, Madagascar

## Abstract

**Background:**

Schistosomiasis is a high-burden parasitic disease and endemic in tropical climates, such as Madagascar. Recently it is emerging in Europe. Chronic infections lead to disabilities including loss in work productivity. Current control strategies focus on school-aged children, thereby systematically excluding adults. The goal to eliminate the disease as a public health problem is aligned with the Sustainable Development Goals agenda, including Universal Health Coverage (UHC). We aim at assessing the distribution of schistosomiasis among adults in Madagascar, in order to promote more targeted public health initiatives against the disease.

**Methods:**

In a cross-sectional study, 1477 blood samples were collected at three primary health-care centers in Madagascar from 03.21 to 01.22. A semi quantitative PCR assay was used to assess the presence of Schistosoma. Positivity rates with 95% Confidence Intervals (CI) were reported by gender and age groups.

**Results:**

Our study population had a balanced gender distribution with 54.2% [CI: 51.7;56.8] of females and a median age of 37 years (IQR: 24-48). Preliminary results show an overall Schistosome positivity rate of 59.5% [CI: 57.0;62.0]. Positivity was higher in males 62.4% [CI: 58.7;66.1], than in females 57.1% [CI: 53.5;60.5]. Highest positivity was seen in the 18-34 year age group with 66.2% [CI: 62.7;69.6]. Lowest positivity of 46.2% [CI: 39.2;53.2] was observed in adults aged over 55 years.

**Conclusions:**

Our preliminary results provide evidence of high positivity rates of schistosomiasis among adults in a highly endemic country. Differently from many studies, males seem more affected than females. The youngest age group, representing the more active workforce of a population, shows a higher positivity rate. Our data suggests that, in the logic of UHC and health as a human right, public health strategies for schistosomiasis need to be re-addressed towards a universal coverage of affected individuals.

**Key messages:**

- Inequalities in current control strategies to fight the tropical disease schistosomiasis might hinder the achievement of disease elimination and universal health coverage goals.
- Further systematically assessment and re-addressing of public health strategies towards universal coverage of affected individuals might help to close gaps in health care and reach the set goals.

